# Dry Gangrene

**Published:** 1830

**Authors:** 


3. *Dry Gangrene.*—The novelty of the case of Dry Gangrene, reported by Dr. Lindsey, in the present number of this Journal, (page 28,) induced us to write to him for some more definite information, particularly whether the symptoms of Dry Gangrene extended to the ulcers on the sacrum and hip. In reply we received the following communication. F.

"Richmond, June 7, 1830.

"Dear Sir—With regard to the ulcers reported in my case as occurring on the sacrum, I am at a loss to say whether symptoms of Dry Gangrene were fully developed or not. On the hip I was more particular in marking the rise and progress of the ulcers than in those of the sacrum. The circumstance of the ulcerative process coming on in the hip without any previous inflammation, might be owing to the extreme exhaustion of the system. As I before stated, a discoloration in the part was first observed, which continued gradually to spread and increase in shade, until of considerable extent, and of a jet black color. To what extent of surface it might have spread before being arrested by the vis medicatrix naturæ, is difficult to say. I found it difficult, even by means of vesicatories and other stimulating dressings to produce a line of separation between the living and the dead parts. The part so soon as it had become black, presented a dry elastic feeling to the sense of touch ; and was very similar to a dry buckskin cushion, stuffed with wool or some soft elastic material. The one on the sacrum had already become abraded when I first observed it, owing no doubt to the patient necessarily remaining so long in the position of recumbency ; it still continued to spread some time, before sufficient tonicity could be produced to arrest it. Before these ulcers made their appearance on the hip, the patient had been turned to lie on that side occasionally, for the purpose of favoring the one on the back. This circumstance ought to be taken into consideration in accounting for the distinction between them and the ulcer on the sacrum. In these points of ulceration on the hip, although there was no abrasion in consequence of the patient lying on the side, it is probable that the ulcerative process was induced by those points being compressed from the weight of the body. In her extreme emaciation, the circulation must of necessity have been very languid, and have required but little pressure to arrest it altogether.

"With sentiments of esteem,

"WM. LINDSEY."

